# Male, Mobile, and Moneyed: Loss to Follow-Up vs. Transfer of Care in an Urban African Antiretroviral Treatment Clinic

**DOI:** 10.1371/journal.pone.0078900

**Published:** 2013-10-24

**Authors:** Kara G. Marson, Kenneth Tapia, Pamela Kohler, Christine J. McGrath, Grace C. John-Stewart, Barbra A. Richardson, Julia W. Njoroge, James N. Kiarie, Samah R. Sakr, Michael H. Chung

**Affiliations:** 1 Department of Global Health, University of Washington, Seattle, Washington, United States of America; 2 Department of Medicine, University of Washington, Seattle, Washington, United States of America; 3 Department of Epidemiology, University of Washington, Seattle, Washington, United States of America; 4 Department of Biostatistics, University of Washington, Seattle, Washington, United States of America; 5 Division of Public Health Sciences, Fred Hutchinson Cancer Research Center, Seattle, Washington, United States of America; 6 University of Nairobi, Nairobi, Kenya; 7 Coptic Hospital, Nairobi, Kenya; Fundacion Huesped, Argentina

## Abstract

**Objectives:**

The purpose of this study was to analyze characteristics, reasons for transferring, and reasons for discontinuing care among patients defined as lost to follow-up (LTFU) from an antiretroviral therapy (ART) clinic in Nairobi, Kenya.

**Design:**

The study used a prospective cohort of patients who participated in a randomized, controlled ART adherence trial between 2006 and 2008.

**Methods:**

Participants were followed from pre-ART clinic enrollment to 18 months after ART initiation, and were defined as LTFU if they failed to return to clinic 4 weeks after their last scheduled visit. Reasons for loss were captured through phone call or home visit. Characteristics of LTFU who transferred care and LTFU who did not transfer were compared to those who remained in clinic using log-binomial regression to estimate risk ratios.

**Results:**

Of 393 enrolled participants, total attrition was 83 (21%), of whom 75 (90%) were successfully traced. Thirty-seven (49%) were alive at tracing and 22 (59%) of these reported having transferred their antiretroviral care. In the final model, transfers were more likely to have salaried employment [Risk Ratio (RR), 2.7; 95% confidence interval (CI), 1.2-6.1; p=0.020)] and pay a higher monthly rent (RR, 5.8; 95% CI, 1.3-25.0; p=0.018) compared to those retained in clinic. LTFU who did not transfer care were three times as likely to be men (RR, 3.1; 95% CI, 1.1-8.1; p=0.028) and nearly 4 times as likely to have a primary education or less (RR, 3.8; 95% CI, 1.3-10.6; p=0.013). Overall, the most common reason for LTFU was moving residence, predominantly due to job loss or change in employment.

**Conclusion:**

A broad definition of LTFU may include those who have transferred their antiretroviral care and thereby overestimate negative effects on ART continuation. Interventions targeting men and considering mobility due to employment may improve retention in urban African ART clinics.

**Clinical Trials:**

The study’s ClinicalTrials.gov identifier is NCT00273780.

## Introduction

Retention in antiretroviral clinics has been identified as a critical component of HIV care. Combination antiretroviral therapy (ART) with three or more antiretroviral drugs reduces both morbidity and mortality from HIV infection [1,2], but its beneficial effects decline when adherence to treatment regimens is inadequate [3]. Patients who miss visits or are lost to follow-up (LTFU) from an ART clinic lack continuous access to their medications and are unable to reach optimal adherence levels necessary for viral suppression [4]. Some studies associate LTFU with other negative outcomes, including treatment failure and death [5-7], and assume the majority of those LTFU represents an interruption in care. 

LTFU is particularly problematic in sub-Saharan Africa, where there are limited resources to track and retain patients in HIV care. While funding such as the President’s Emergency Plan for AIDS Relief (PEPFAR) has increased global access to ART over the past decade [8], growing patient numbers have challenged the ability of many clinics to successfully track and retain individuals who are at risk for LTFU [9,10]. Two systematic reviews of ART clinic retention in sub-Saharan Africa indicate that attrition is approximately 20% at six months after ART initiation, over half of which is attributed to LTFU [11,12]. 

Few studies have examined the timing and reasons for LTFU, and none has analyzed the characteristics of LTFU who have transferred their HIV care elsewhere and differentiated them from those LTFU who did not transfer their care. This manuscript describes the correlates and reasons for LTFU with and without subsequent transfer of care from an ART clinic in Nairobi, Kenya.

## Methods

### Ethics Statement

The study protocol was reviewed and approved by the institutional review board at the University of Washington (Seattle, USA) and the Ethics and Research Committee at Kenyatta National Hospital (Nairobi, Kenya). 

This study was conducted at the Coptic Hope Center for Infectious Diseases, a PEPFAR-funded ART treatment facility in Nairobi, Kenya [13]. Jointly established by the Coptic Orthodox Mission and the University of Washington, the Hope Center offers free ART and comprehensive clinical care to HIV-positive adults and children in accordance to Kenyan Ministry of Health national guidelines [14]. Patients who initiate ART at the Hope Center receive clinical consultations every one to three months, CD4 testing every six months, free treatment for opportunistic infections, and psychosocial and nutritional support.

In 2006, a prospective cohort of patients from the Hope Center was enrolled in a randomized controlled trial (RCT) that compared ART adherence between those who received educational counseling, those who carried a pocket alarm device, those who received both, and those who received neither intervention [13]. Hope Center patients were eligible for the trial if they were at least 18 years of age, were ART naïve and eligible to initiate treatment based on Kenyan national guidelines, agreed to home visits by study staff, planned to stay in Nairobi for at least two years, and provided written informed consent. Sociodemographic variables, CD4 count, and plasma HIV-1 RNA viral load were collected at study enrollment, as well as personal phone numbers, alternative contact phone numbers for friends and family, home addresses, local bus routes, and neighborhood landmarks. The participant’s home was visited at enrollment by a community health worker, and geographic coordinates were obtained with a handheld GPS device to facilitate future tracing and to calculate distance from home to clinic. The cost of traveling to the clinic from home was self-reported. Participants from this trial were included in this secondary analysis to determine the correlates and reasons for LTFU with and without transfer of care.

Trial participants were followed from the time of initial enrollment at Hope Center to 18 months after initiating ART, and were scheduled to return monthly to the study pharmacy to pick up their antiretroviral medications. No financial incentives or travel reimbursement were given for visits to the pharmacy and clinic. Participants who were more than four weeks late for their scheduled monthly pharmacy visit (approximately eight weeks from their last documented attendance) were called twice on the phone by the study receptionist based on the preferred contacts given at enrollment and were visited at home by a community health worker. Participants were defined as LTFU if they failed to return to clinic after these interventions. Reasons for loss and whether the participants transferred their care were self-reported or gathered from household members and neighbors via phone or home visit.

LTFU was retrospectively classified into one of two outcomes: “transfer of care” or “no care,” based on whether the source reported that the participant was receiving care at an alternative HIV treatment facility. The referent group for comparisons was comprised of those patients who remained in care at the Hope Center during the 18-month follow-up period. Employment, education level, monthly rent, and use of private vs. communal flush toilet or pit latrine were evaluated as proxy indicators of socio-economic-status (SES), as is consistent with household amenity SES proxies that other studies in sub-Saharan Africa have used [15]. 

Baseline characteristics for the “transfer of care” and the “no further care” groups were compared to the referent group of those who remained in care using log-binomial regression to estimate risk ratios. All analyses were adjusted for the factorial design of the trial, and variables with individual category or overall group significance p<0.10 were combined in a preliminary multivariate model. Variables significant at p<0.10 were retained in the final multivariate model. All quantitative analyses were two-sided and conducted using Stata Intercooled v11 (StataCorp., College Station, TX, USA).

## Results

**Figure 1:**
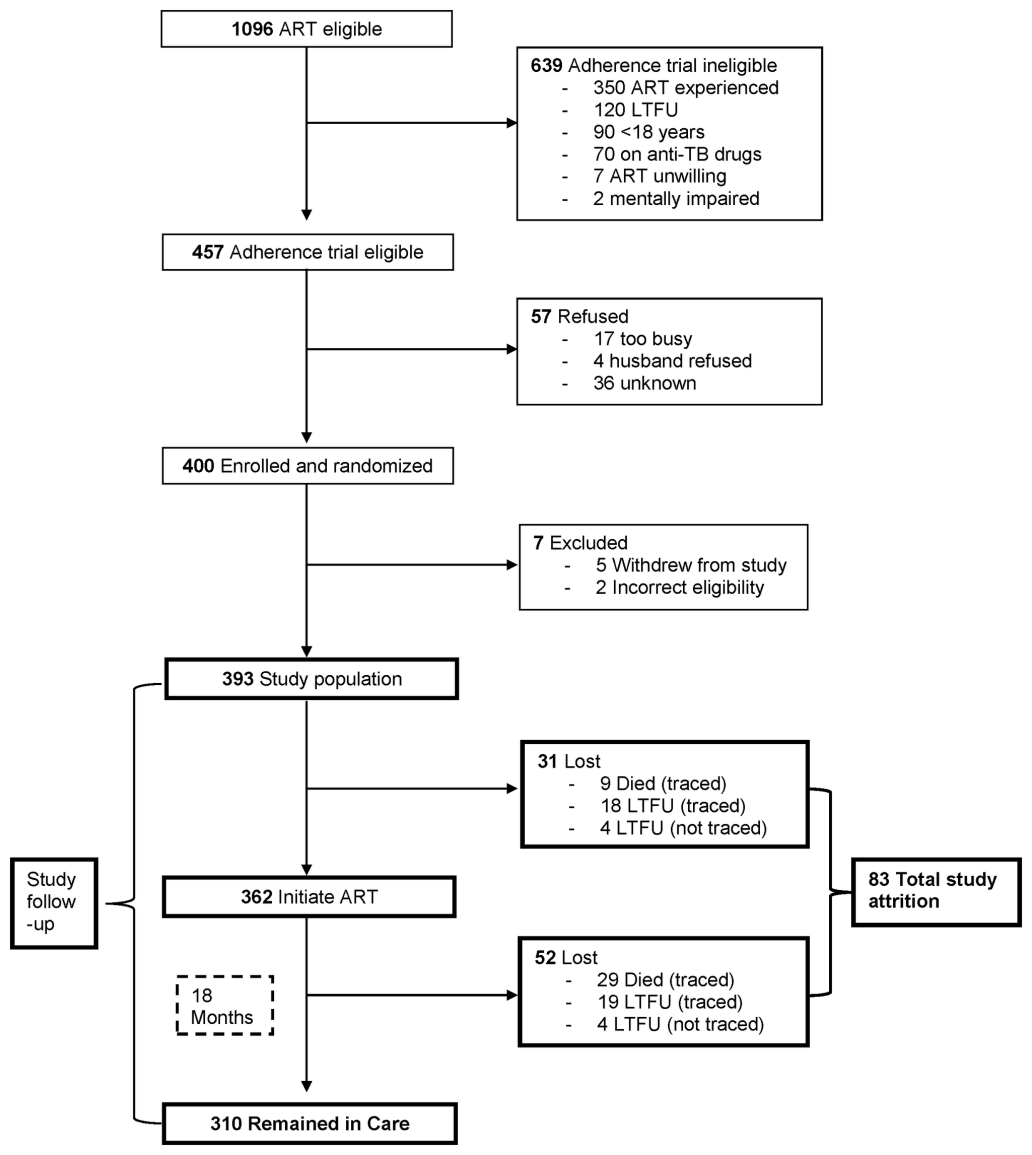
Trial profile of ART eligible patients screened at Coptic Hope Center.

Between May and November 2006, 1096 patients at the Hope Center were screened and advised to initiate ART, of whom 457 (42%) were ART naïve and eligible to participate in the adherence trial. Of the trial-eligible patients, 400 (88%) were enrolled and randomized (Figure 1). Seven participants were excluded from these analyses due to incorrect trial eligibility or withdrawal from the RCT. Among the 393 remaining participants, the median age was 36 years [Interquartile Range (IQR), 31-42], 66% were female, and the median monthly rent was US$27 (IQR, 11-53). Sixty-seven percent (263) were employed at baseline (30% had salaried employment, 23% were self-employed in jobs such as salon or shop-owners, and 14% were casual laborers in jobs such as construction and house-cleaning).

**Figure 2:**
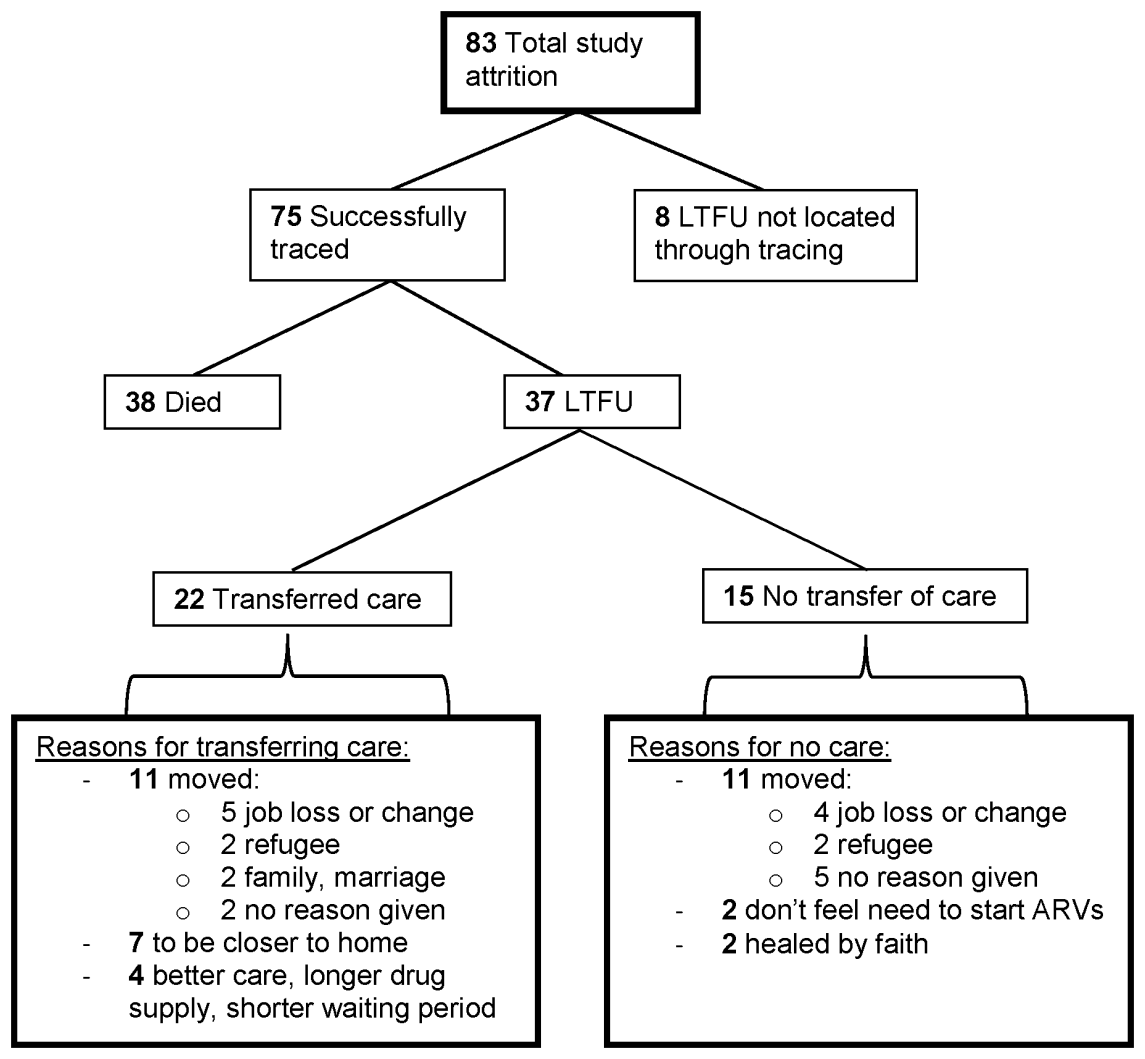
Attrition outcomes and reasons for loss.

Three hundred and ten participants completed 18 months of follow-up after ART initiation and 83 participants (21%) failed to return to clinic. Thirty one participants never returned to the Hope Center for ART initiation, and 52 participants returned for a median of 4.3 months (IQR, 1-8) before subsequently failing to return (Figure 1). Among the 83 participants who did not return to clinic, 75 (90%) were successfully traced and 8 (10%) were not located (Figure 2). Of the 75 traced participants, 38 (51%) had died and 37 (49%) were alive but LTFU. Eighteen (49%) of the 37 LTFU were lost prior to ART initiation, and 19 (51%) were lost after. 

Of the 37 LTFU who were traced, reasons for loss were obtained from 31 (84%) patients directly through self-report, and from 6 patients (16%) indirectly through family members, friends, or neighbors. Twenty two (59%) of the 37 had transferred their HIV care to another ART clinic and 15 (41%) did not transfer their HIV care elsewhere. Among the 22 participants who had transferred care, 11 (50%) transferred due to moving out of the area: 5 lost or changed jobs, 2 were refugees, 2 moved for family or marriage, and 2 did not give a further explanation. A further 7 (32%) participants had transferred their care to an HIV clinic closer to home, and 4 (18%) sought better clinical care elsewhere (Figure 2). Ten (45%) had transferred prior to ART initiation and 12 (55%) had transferred after. 

LTFU participants who transferred care were more likely to have salaried employment (55% vs. 29%; p=0.014), pay a higher rent of >US$45 (64% vs. 30%; p=0.016), and use a flush toilet instead of pit latrine (68% vs. 45%; p=0.047) than those who remained in care at the Hope Center (Table 1). In the final model, participants who transferred care were more than twice as likely to be salaried [adjusted Risk Ratio (aRR), 2.7; 95% Confidence Interval (CI), 1.2-6.1; p=0.020] and nearly six times as likely to pay a monthly rent greater than US$ 45 (aRR, 5.8; 95% CI, 1.3-25.0; p=0.018) compared to those who remained in care (Table 2).

**Table 1:** Baseline characteristics of LTFU who transferred care and LTFU who received no further care vs. **those who remained in care**.

**Category**		**Remained in Care**	**Transferred Care**	P-value*	**No Care**	P-value*
		**(N=310)**	**(N=22)**		**(N=15)**	
		**n (%)**	**n (%)**		**n (%)**	
		**median (IQR)**	**median (IQR)**		**median (IQR)**	
Age						
	<30	49 (16)	7 (32)		4 (27)	
	30-39	155 (50)	10 (46)	0.161	6 (40)	0.29
	40-49	81 (26)	4 (18)	0.107	4 (27)	0.49
	>50	25 (8)	1 (4)	0.22	1 (7)	0.49
				(0.28)		(0.74)
Male		105 (34)	4 (18)	0.138	9 (60)	0.043
Married		154 (50)	7 (32)	0.124	7 (47)	0.83
Salaried employment		90 (29)	12 (55)	0.014	3 (21)	0.56
< Primary education		108 (35)	8 (36)	0.88	9 (60)	0.062
Number in household						
	1-2	82 (26)	9 (41)		7 (47)	
	3-4	120 (39)	4 (18)	0.87	6 (40)	0.26
	5-6	67 (22)	5 (23)	0.134	1 (7)	0.50
	>7	41 (13)	4 (18)	0.68	1 (7)	0.73
				(0.27)		(0.32)
Toilet						
	Pit latrine	169 (55)	7 (32)		9 (60)	
	Flush toilet	141 (45)	15 (68)	0.047	6 (40)	0.70
Share a toilet		170 (55)	10 (45)	0.42	11 (73)	0.168
Monthly rent (US$)						
	<15	89 (33)	2 (9)		2 (13)	
	15-29	62 (24)	4 (18)	0.27	4 (27)	0.30
	30-45	33 (13)	2 (9)	0.32	4 (27)	0.048
	>45	79 (30)	14 (64)	0.016	5 (33)	0.22
				(0.058)		(0.25)
Cost from clinic to house (US$)						
	<25	28 (9)	2 (9)		0 (0)	
	25-49	86 (28)	8 (36)	0.68	7 (47)	0.99
	50-74	144 (46)	10 (46)	0.95	8 (53)	0.99
	>75	52 (17)	2 (9)	0.54	0 (0)	1.00
				(0.66)		(0.86)
Distance from clinic to house (Km)						
	<5	40 (14)	2 (15)		0 (0)	
	5-9	85 (29)	6 (46)	0.73	7 (64)	0.99
	10-15	84 (29)	4 (31)	0.89	3 (27)	0.99
	>15	82 (28)	1 (8)	0.24	1 (9)	0.99
				(0.45)		(0.26)
Plasma HIV viral load (log_10_)						
	<5.0	51 (17)	5 (24)		5 (36)	
	5.0-5.4	70 (23)	5 (24)	0.80	3 (21)	0.40
	5.5-5.9	104 (34)	5 (24)	0.35	3 (21)	0.138
	>6.0	83 (27)	6 (39)	0.80	3 (21)	0.29
				(0.81)		(0.47)
CD4 cell count (cells/mm^3^)						
	<60	69 (22)	5 (23)		2 (13)	
	60-119	81 (26)	9 (41)	0.40	4 (27)	0.49
	120-179	81 (26)	3 (14)	0.30	2 (13)	0.82
	>180	79 (26)	5 (23)	0.76	7 (47)	0.21
				(0.28)		(0.35)

* P-values are adjusted for study design using log-binomial regression, and are for comparison with the group retained in care

**Table 2:** Multivariate analyses of LTFU who transferred care vs. **those who remained in care**.

**Category**		**Risk Ratio (95% Confidence Interval)**	**P-value**
Salaried employment		2.7 (1.2-6.1)	0.020
Monthly rent (US$)			
	<15	Ref.	
	15-29	2.4 (0.5-12.9)	0.29
	30-45	2.2 (0.3-15.0)	0.42
	>45	5.8 (1.3-25.0)	0.018
			(0.052)

Among the 15 LTFU who did not transfer their care, 11 (73%) had moved: 4 lost or changed jobs, 2 were refugees, and 5 moved without citing a specific reason. Two (15%) participants felt they did not need ART, and 2 (15%) reported having been healed by faith (Figure 2). Of the LTFU who had not transferred, 8 (53%) were lost prior to ART initiation and 7 (47%) were lost after. LTFU participants who did not transfer care were more likely to be male (60% vs. 34%; p=0.043), have a primary education or less (60% vs. 35%; p=0.062), and have a monthly rent of US$30-45 (27% vs. 13%; p=0.048) than those who were retained in care (Table 1). In the final model, LTFU who did not transfer their care were more than three times as likely to be men (aRR, 3.1; 95% CI, 1.1-8.1; p=0.028), nearly 4 times as likely to have a primary education or less (aRR, 3.8; 95% CI, 1.3-10.6; p=0.013), and over 7 times as likely to have a low average monthly rent between US$30-45 (aRR, 7.2; 95% CI, 1.4-37.6; p=0.019) (Table 3). 

**Table 3:** Multivariate analyses of LTFU who received no further care vs. **those who remained in care**.

**Category**		**Risk Ratio (95% Confidence Interval)**	**P-value**
Male		3.1 (1.1-8.1)	0.028
< Primary education		3.8 (1.3-10.6)	0.013
Monthly rent (US$)			
	<15	Ref.	
	15-29	2.1 (0.4-10.9)	0.37
	30-45	7.2 (1.4-37.6)	0.019
	>45	3.8 (0.8-18.6)	0.097
			(0.096)

Clinical disease indicators CD4 count and viral load did not differ significantly between LTFU and retained patients or between those who transferred care and retained patients.

## Discussion

In this study, we prospectively assessed clinic attrition among HIV-positive patients attending an antiretroviral treatment clinic in Nairobi, Kenya. Half of all LTFU in this cohort occurred prior to ART initiation and successful transfer of HIV care comprised the majority of those who had been defined as lost. Compared to those who remained in care, those who transferred HIV care were of higher socioeconomic status (SES). Those who did not transfer care after being lost were significantly more likely to be men or be less educated. Overall, moving one’s home was the most commonly cited reason for being LTFU. 

LTFU in this study was defined as not having come to clinic four weeks after the last scheduled appointment. On further examination, most of these individuals who were identified as LTFU were found to have enrolled in HIV care elsewhere. The proportion of transfers among LTFU in our study (59%) is higher than that seen in related studies (20-48%) [16-21]. This may be due to the fact that untraced LTFU and mortalities were excluded from this analysis, and that our study had a very high percentage (90%) of successfully traced LTFU. Our findings suggest that “LTFU” can be an imprecise criterion that does not exclude successful transfer of care and may incorrectly include those who do not necessarily experience treatment interruption and subsequent risk of antiretroviral failure. It is important to develop systems of communication and referral between health centers to better identify, characterize and document successful transfers, thus making available resources to target those patients who are truly lost from care. 

Those who transferred care in our study reported a higher monthly rent than that of retained participants, as well as other significantly higher SES indicators salaried employment and use of a flush toilet. Higher SES may reflect greater financial means to explore multiple public and private clinic options prior to committing to an ART clinic, especially in an urban setting such as Nairobi where HIV treatment clinics are abundant and offer a variety of free ancillary support incentives to join. The few studies that have examined correlates of HIV care transfer in ART patients have focused primarily on more rural and lower SES populations. In these cohorts, cost of transportation was the most commonly cited reason for transferring care [20,22,23]. In contrast, our patient population in Nairobi, the capital of Kenya, had a greater cross-section of patients with higher SES [24]. As a result, our study may have identified an urban African population with increased financial means to compare and choose between multiple care options available in the city. Programmatic efforts to increase retention should note heterogeneous motivations, even within developing countries, and utilize interventions tailored to the needs of their specific population. 

Among those who did not transfer their HIV care after leaving the clinic, male gender lower education, and monthly rent were significantly predictive, with men being over three times as likely not to be in care as women. The association between male gender and overall LTFU is well-documented in ART retention literature [25-32], and becomes even more pronounced in the context of our finding that the majority of women who were categorized as LTFU had successfully transferred care elsewhere. Men are less likely to be adherent to their medications [33] or achieve favorable clinical outcomes in other chronic care medical conditions as well [34]. Our findings support the concept that HIV-positive men need targeted maintenance and retention interventions. While our study did not assess substance use or advanced WHO stage at presentation to HIV care, both of which are typically more common among men, such factors may underlie the gender difference of LTFU and failure to transfer care and should be considered in retention interventions. Men have also been seen to have higher risk for treatment disruption during situations of political instability or violence [35]. Family-based approaches, male peer-educators, and respected male public figures advocating for behavior change may decrease loss from ART clinics among men [26,32]. Lower education and monthly rent among those who were LTFU without transfer of care contrast with our findings of higher SES among LTFU who did transfer, and demonstrates the association of higher SES with better clinical care follow-up. 

Among all LTFU in this cohort, moving was the most common reason for loss, the majority of which was due to job loss or change in employment. This reflects the substantial impact of employment on geographic mobility in this urban African population. Uneven economic development in resource-limited settings destabilizes populations, promotes migration [36,37], and may thereby increase LTFU from HIV clinics. The majority of the population in Africa is concentrated in less than a quarter of the land surface, resulting in the social and economic isolation of many rural communities [38]. Population growth in cities is often fed by rural to urban migration based on the expectation of increased economic opportunities [39], yet high levels of unemployment may perpetuate the mobility of job seekers, driving them to work and reside far from their ART clinic. Our findings also showed that half of all of loss occurred before ART initiation, indicating the pre-ART period as one in which retention interventions are particularly important [40,41]. Links with social workers and strategies to address mobility and employment before ART initiation may therefore enhance retention efforts and reduce overall treatment failure. 

While other studies in sub-Saharan Africa have examined transfer of care as an outcome in addition to retention, mortality, and LTFU [16-19,23,28,31], ours was unique in determining correlates of LTFUs who did and did not transfer their care elsewhere. A major strength of this study was the high percentage of participation among study-eligible patients at the Coptic Hope Center, indicating a fairly representative sample of the population seeking care at this ART clinic. Additionally, the original study from which this data was derived did not engage staff members in extensive patient follow-up procedures until eight weeks had passed from their last documented visit to the clinic pharmacy. In this way, the data presented here may represent a more realistic picture of LTFU from an ART clinic compared to trials engaging earlier and more rigorous retention methods. Our study also benefited from a high response rate from the LTFU patients themselves and timely assessment of qualitative reasons for loss. 

There are several study weaknesses. One is that our tracing and interviewing procedures did not probe for more in-depth responses to LTFU. A more rigorous implementation of qualitative data collection may have elicited additional reasons for LTFU that have been commonly cited by other studies. These include stigma/discrimination, lack of support, and fear of disclosure [18,42-45] and poor patient-provider relationships or clinical outcomes [23,44,46]. At the same time, many of these reasons may have been less of an issue at the Hope Center, the HIV clinic from which participants were enrolled. Supported by PEPFAR and the Coptic Christian Mission, the Hope Center provides free comprehensive medical care, counseling, nutritional and social work services in addition to ART, was observed to have delivered quality HIV care under political duress, and has patient retention rates that are higher than surrounding HIV clinics [24,35]. Secondly, transfer of care was based on self-report and we were not able to verify transfer or extent of care directly with the new HIV clinic. Subsequently, it is also not known if these patients who transferred remained in care or continued ART at the other site. Finally, although the study elicited in-depth qualitative information on the reasons for loss, the sample of LTFU patients was relatively small, suggesting that there may be limits to the generalizability of these findings. 

This study examined patients lost to an urban ART clinic in sub-Saharan Africa and described reasons for transfer and loss from care. Our findings suggest that interventions focused on men and integration of employment counseling or job networks within comprehensive HIV care will enhance clinic retention. Programs that can identify and document clinic transfers more accurately will improve retention and save resources by being able to target those who are truly lost to care. 
